# Supplementary figures and images for: Risk of cancer with angiotensin-receptor blockers increases with increasing cumulative exposure: Meta-regression analysis of randomized trials
Source: PLoS One. 2022 Mar 2;17(3):e0263461. doi: 10.1371/journal.pone.0263461 (PMC8890666; doi:10.1371/journal.pone.0263461)

**
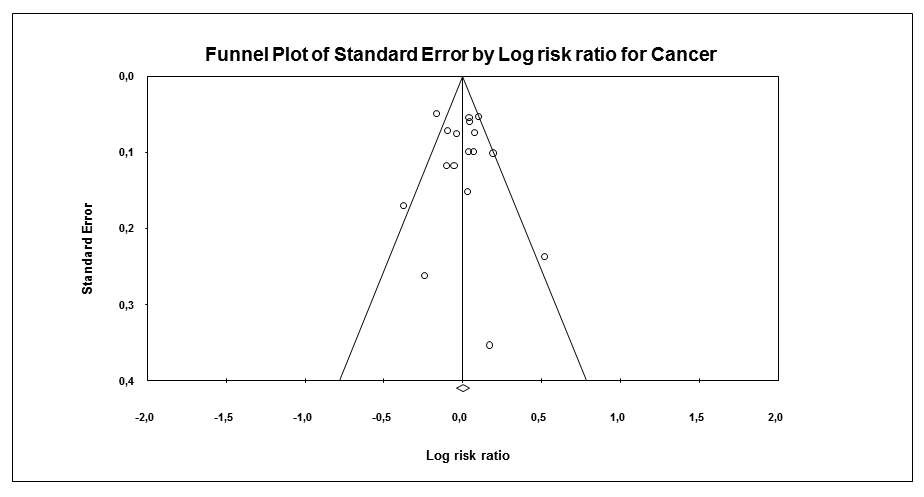
**

Supplement: S1 Fig — (DOCX) [file pone.0263461.s001.docx]

**
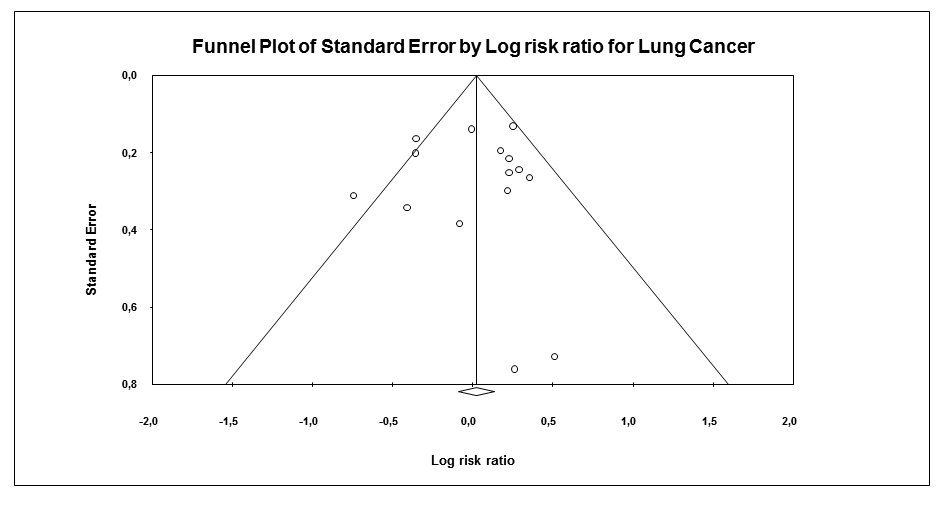
**

Supplement: S2 Fig — (DOCX) [file pone.0263461.s002.docx]
