# Supplementary material for: Risk of cancer with angiotensin-receptor blockers increases with increasing cumulative exposure: Meta-regression analysis of randomized trials
Source: PLoS One. 2022 Mar 2;17(3):e0263461. doi: 10.1371/journal.pone.0263461 (PMC8890666; doi:10.1371/journal.pone.0263461)
Supplement: S1 Table — (DOCX) [file pone.0263461.s003.docx]

| **Study Name** | **Total n** | **Average age (years)** | **Men, n (%)** | **Smokers, n (%)** | **Background ACE-inhibitor treatment (%)** |
| --- | --- | --- | --- | --- | --- |
| ONTARGET | 25,620 | 66.4 | 18,789 (73.3) | 16,501 (64.4) | As randomized |
| TRANSCEND | 5,926 | 66.9 | 3,379 (57.0) | 3,138 (53.3) | None |
| PROFESS | 20,332 | 66.1 | 13,022 (64.1) | 11,660 (57.3) | 36.5 |
| I-PRESERVE | 4,128 | 72.0 | 1,637 (39.7) | NA | 25 |
| ACTIVE-I | 9,016 | 69.5 | 5,475 (60.7) | 4,505 (50.0) | 60.4 |
| IDNT | 1,715 | 59.3 | 1,140 (66.5) | NA | None |
| VAL-HEFT | 5,010 | 62.7 | 4,005 (79.9) | NA | 93 |
| VALIANT | 14,703 | 64.8 | 10,133 (68.9) | 4,664 (31.7) | As randomized |
| VALUE | 15,245 | 67.3 | 8,777 (57.6) | 24% ^a^ | None |
| NAVIGATOR | 9,306 | 63.7 | 4,711 (50.6) | NA | 7.3 |
| CHARM-OVERALL | 7,599 | 65.9 | 5,199 (68.4) | NA | CHARM-Added: 100  CHARM-Alternative: None  CHARM-Preserved: 19.1 |
| SCOPE | 4,964 | 76.4 | 1,780 (35.9) | 432 (8.7) | None |
| TROPHY | 772 | 48.5 | 460 (59.6) | NA | None |
| DIRECT (all) | 5,231 | 40,3 | 2844 (54.4) | 1637 (31.3) | None |
| LIFE | 9,193 | 66.9 | 4230 (46.0) | 1,499 (16.3) | None |

^a^ Only percentage available. Absolute number of smokers not available
