# Supplementary material for: Risk of cancer with angiotensin-receptor blockers increases with increasing cumulative exposure: Meta-regression analysis of randomized trials
Source: PLoS One. 2022 Mar 2;17(3):e0263461. doi: 10.1371/journal.pone.0263461 (PMC8890666; doi:10.1371/journal.pone.0263461)
Supplement: S2 Table — (DOCX) [file pone.0263461.s004.docx]

**S2 Table. Sensitivity analyses with the one-study out method**

| **Dggf Metaregression for cumulative exposure to ARBs and log risk ratio of cancer** | | | | |
| --- | --- | --- | --- | --- |
| **Excluded study** | **Slope (95% CI)**  **(fixed effect)** | **P value** | | |
|  |  | **Fixed effect** | **Mixed effects - method of moments** | **Mixed effects – unrestricted**  **maximum likelihood** |
| ACTIVE- I | 0.07 (0.03-0.12) | <0.001 | 0.003 | 0.001 |
| CHARM-OVERALL | 0.07 (0.03-0.11) | <0.001 | 0.007 | 0.002 |
| DIRECT (all) | 0.07 (0.03-0.11) | <0.001 | 0.008 | 0.005 |
| IDNT | 0.07 (0.03-0.11) | <0.001 | 0.009 | 0.003 |
| I-PRESERVE | 0.07 (0.03-0.11) | <0.001 | 0.009 | 0.003 |
| LIFE | 0.07 (0.03-0.11) | <0.001 | 0.01 | 0.006 |
| NAVIGATOR | 0.08 (0.04-0.12) | <0.001 | 0.003 | <0.001 |
| ONTARGET | 0.08 (0.03-0.14) | 0.005 | 0.03 | 0.02 |
| PROFESS | 0.07 (0.03-0.11) | <0.001 | 0.01 | 0.005 |
| SCOPE | 0.08 (0.04-0.12) | <0.001 | 0.002 | <0.001 |
| TRANSCEND | 0.06 (0.02-0.10) | 0.002 | 0.02 | 0.009 |
| TROPHY | 0.07 (0.03-0.11) | <0.001 | 0.006 | 0.002 |
| VAL-HEFT | 0.07 (0.03-0.11) | <0.001 | 0.01 | 0.005 |
| VALIANT | 0.07 (0.03-0.11) | 0.001 | 0.02 | 0.009 |
| VALUE | 0.05 (0.005-0.09) | 0.03 | 0.04 | 0.03 |
| **Dggf Metaregression for cumulative exposure to ARBs and log risk ratio of cancer** | | | | |
| **Excluded study** | **Slope (95% CI)**  **(fixed effect)** | **P value** | | |
|  |  | **Fixed effect** | **Mixed effects - method of moments** | **Mixed effects – unrestricted**  **maximum likelihood** |
| ACTIVE- I | 0.16 (0.05-0.27) | 0.004 | 0.02 | 0.01 |
| CHARM-OVERALL | 0.17 (0.07-0.29) | 0.002 | 0.007 | 0.004 |
| DIRECT (all) | 0.16 (0.05-0.27) | 0.004 | 0.02 | 0.01 |
| IDNT | 0.16 (0.05-0.27) | 0.003 | 0.02 | 0.008 |
| I-PRESERVE | 0.16 (0.05-0.27) | 0.004 | 0.01 | 0.01 |
| LIFE | 0.16 (0.05-0.27) | 0.005 | 0.03 | 0.02 |
| NAVIGATOR | 0.14 (0.03-0.26) | 0.01 | 0.03 | 0.02 |
| ONTARGET | 0.26 (0.09-0.43) | 0.003 | 0.01 | 0.006 |
| PROFESS | 0.19 (0.07-0.30) | 0.001 | 0.004 | 0.001 |
| SCOPE | 0.17 (0.06-0.28) | 0.003 | 0.02 | 0.009 |
| TRANSCEND | 0.15 (0.04-0.27) | 0.007 | 0.03 | 0.02 |
| VAL-HEFT | 0.18 (0.07-0.30) | 0.001 | 0.005 | 0.003 |
| VALIANT | 0.13 (0.02-0.25) | 0.02 | 0.0499 | 0.04 |
| VALUE | 0.13 (0.006-0.25) | 0.04 | 0.06 | 0.049 |
